# Classification of Partial Discharge Measured under Different Levels of Noise Contamination

**DOI:** 10.1371/journal.pone.0170111

**Published:** 2017-01-13

**Authors:** Wong Jee Keen Raymond, Hazlee Azil Illias, Ab Halim Abu Bakar

**Affiliations:** 1 Department of Electrical and Electronic Engineering, Faculty of Engineering and Built Environment, Tunku Abdul Rahman University College, Kuala Lumpur, Malaysia; 2 Department of Electrical Engineering, Faculty of Engineering, University of Malaya, Kuala Lumpur, Malaysia; 3 UM Power Energy Dedicated Advanced Centre (UMPEDAC), Level 4, Wisma R&D UM, University of Malaya, Jalan Pantai Baharu, Kuala Lumpur, Malaysia; Tianjin University, CHINA

## Abstract

Cable joint insulation breakdown may cause a huge loss to power companies. Therefore, it is vital to diagnose the insulation quality to detect early signs of insulation failure. It is well known that there is a correlation between Partial discharge (PD) and the insulation quality. Although many works have been done on PD pattern recognition, it is usually performed in a noise free environment. Also, works on PD pattern recognition in actual cable joint are less likely to be found in literature. Therefore, in this work, classifications of actual cable joint defect types from partial discharge data contaminated by noise were performed. Five cross-linked polyethylene (XLPE) cable joints with artificially created defects were prepared based on the defects commonly encountered on site. Three different types of input feature were extracted from the PD pattern under artificially created noisy environment. These include statistical features, fractal features and principal component analysis (PCA) features. These input features were used to train the classifiers to classify each PD defect types. Classifications were performed using three different artificial intelligence classifiers, which include Artificial Neural Networks (ANN), Adaptive Neuro-Fuzzy Inference System (ANFIS) and Support Vector Machine (SVM). It was found that the classification accuracy decreases with higher noise level but PCA features used in SVM and ANN showed the strongest tolerance against noise contamination.

## 1. Introduction

Important power system equipment such as gas insulated switchgear, transformers and high voltage (HV) power cables operation life span is highly dependent on the insulation quality. They will be permanently damaged if insulation breakdown occurs. Failure in any part of the power system will be detrimental to energy generation and transmission companies. Hence, it is extremely important to check the insulation quality frequently. Partial discharge (PD) measurement is globally accepted as a useful diagnostic technique with the ability to assess insulation material for its condition [1]. According to the IEC 60270 standard, PD is defined as “localized electrical discharge that only partially bridges the insulation between conductors.” [2]. PD is repetitive in nature and able to spread across the dielectric material. PD intensifies existing insulation impairment and causes steady deterioration of insulating quality, ultimately leading to electrical breakdown, hazard to personnel, environmental damage and costly equipment failures [3]. Since PD events may lead to disastrous results with both safety and financial consequences, detection of PD events are used as a key method in insulation system condition monitoring [4].

PD classification is of interest because of the relationship between the PD activity and the dielectric materials aging process. Since each defect has a unique deterioration behavior, it is important to recognize the association between the PD patterns and the defect type in order to determine the insulation quality. PD pattern recognition is crucial in determining substantial risk of an imminent insulation breakdown and consequently whether the current component requires servicing and replacement or not [5]. Many works have been performed on PD classification in various power system equipment, such as gas insulated switchgears and substations [6, 7], power cables [8, 9] and transformers [10, 11]. Commonly used classifiers include neural networks [7, 12], fuzzy logic [13, 14] and support vector machines [15, 16].

PD has a group of unique discriminatory attributes, which allows them to be recognized. In order to perform PD classification, it is necessary to choose which discriminatory features to be extracted and which feature extraction method to be used [17]. The purpose of feature extraction is to extract meaningful input feature from the unprocessed PD data to represent the PD pattern associated with a specific defect [18]. These extracted features are used as input of the classifier during the training process. Feature extraction also helps to reduce the size of raw PD data for quicker and simpler handling. PD classification requires some sort of data reduction method, such as reducing the matrix size [19]. This is due to unprocessed PD data which may contain thousands to millions of individual pulses are too huge to be used as input to the classifiers as it will drastically increase the training time and cripple the performance of the classifier [20, 21].

Most of PD classification works were performed in lab environment and under noise free environment. However, in reality, on site PD measurement suffers from lower detection sensitivity due to the interference of external noises [22]. PD measurement often faces interference caused by radio transmissions, power electronics components, random noise from switching, lightning, arcing, harmonics and interferences from ground connections [23]. A lot of research work has been performed on denoising PD data. One of the methods involves setting a threshold and ignoring PD data that are 10% of the maximum PD amplitude. However, it was found to be insufficient as high threshold level might neglect real PD pulses with low magnitude and low threshold level will include noise [24, 25]. Using the mean square error as a benchmark to compare 28 types of denoising technique, wavelet based denoising was found to be the best with good signal to noise ratio [26]. Numerous research works have also used wavelet transform for denoising purposes, especially the Daubechies wavelet, which is capable of detecting high frequency, fast decaying, short duration, and low amplitude signals [27, 28].

PD denoising techniques have improved over the years. However, a perfect and universal denoising standard has yet to be achieved. Therefore, some researchers have included artificial noise signals into PD data before evaluating the PD classification model in order to replicate the practical scenario. For example, adding evenly distributed random number to phase and charge of PD data [1, 29], adding white noise with zero mean and fluctuating power [23], including random numbers with various standard deviation and zero mean [30] and merging randomly distributed noise that are within 10 to 30% of the test data [31–37]. The effect of adding noise are summarized as follows; in [1], the accuracy of ANN reduces from 79% under noise free condition to 42.2% with 10% added noise, in [36, 37], the accuracy of ANN reduces from 100% under noise free condition to 80% with 30% random noise, in [34, 38], when 30% noise was introduced, the accuracy of ANN reduces from 100% to between 70 and 80% depending on the input feature used. In [31], ANN accuracy reduces from 93.7% to 83.3%.

However, artificially generated noise using software, as applied in previous works may not represent real world scenario. Therefore, in this work, classifications of cable joint defect types from PD measurement under noisy environment were performed. Real life noise obtained from ground interference instead of software generated noise as commonly used in past works was used in this work. This is a better representation of noise encountered on-site. Five cross-linked polyethylene (XLPE) cable joints with artificially created defects were prepared. After PD measurement was performed on each cable joint sample, different input features were extracted from the PD pattern under artificially created noisy environment. These include statistical features, fractal features and principal component analysis (PCA) features. The input features were used to train the classifiers to classify the PD defect types using Artificial Neural Networks (ANN), Adaptive Neuro-Fuzzy Inference System (ANFIS) and Support Vector Machine (SVM). At the end of the work, comparison between different combinations of feature extraction and classifiers was made to determine which method has the highest classification accuracy result or highest noise tolerance.

Time series analysis is a very useful tool. The directed weighted complex network method can be used to distinguish and characterize different dynamical regimes associated with unstable periodic orbits from time series signals [39]. For nonlinear dynamic behavior in gas—liquid two-phase flow, the multivariate weighted complex network can be used [40]. On the other hand, multivariate pseudo Wigner distribution allows uncovering local flow behavior revealing different oil—water flow patterns [41]. Gao et al. proposed a multiscale limited penetrable horizontal visibility graph to analyze nonlinear time series [42] and then developed a novel AOK-TFR based visibility graph to classify epileptic EEG signals [43].

The rest of the paper is organized as follows. Section 2 describes the test samples preparation. In section 3, the measurement setup is outlined. Section 4 elaborates on the feature extraction methods used. The classifiers used are explained in Section 5, followed by the results in Section 6. Lastly, the conclusion can be found at Section 7.

## 2. Sample Preparation

Five 11 kV XLPE cable joint with different artificial defects were prepared. The total length of each cable sample is 3 meters with a cable joint located in the centre. The details of the defect nature of all cable joint samples are shown in Table 1.

**Table 1:** XLPE cable joint defect samples.

Sample	XLPE Cable Joint Defect
C1	Insulation incision defect
C2	Axial direction shift
C3	Semiconductor layer tip
C4	Metal particle on XLPE
C5	Semiconductor layer air gap

Insulation incision defect was prepared by creating a shallow cut at the XLPE surface using a blade. Axial direction shift defect was prepared by inserting the cable at a shifted angle. Semiconductor layer tip defect was made by making numerous sharp edges at the semiconductor tip. Metal particle on XLPE defect was prepared by spreading metal bits on the XLPE layer while semiconductor layer air gap defect was prepared by wrapping insulation tape at the boarder of the semiconductor layer and XLPE insulation layer. All cable defects were created on the cable prior to the cable joints installation.

## 3. PD Measurement Setup

**Fig 1:**
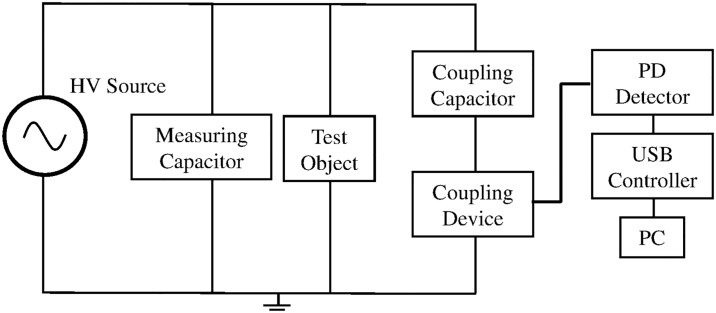
Block diagram of PD measurement setup.

Fig 1 shows the block diagram of the PD measurement system that was used in this work. The measurement setup comprises of a step-up transformer that serves as a high voltage source, a coupling device, a test object, a coupling and measuring capacitor, a USB controller and a PD detector connected to a personal computer. A personal computer (PC) was used to store the measured PD data. A commercial PD detector MPD600 from Omicron was used in this work.

All measurements were performed at 9 kV, which is slightly less than the 11 kV rated voltage of the cable. This is because operating at higher applied voltage will significantly increase the likelihood of insulation breakdown at cable joint defect, which will cause permanent damage to the test sample. Each cable joint was energized to 9 kV and allowed to be idle for 1 hour for the PD to reach a steady state before PD measurement was taken. Each PD measurement was taken for 1 minute with a time gap of 15 seconds between every measurement. A total of 100 measurements were performed on each cable joint sample. The results are shown in term of phase resolved partial discharge (PRPD) patterns, a 3D plot with phase, charge magnitude and pulse count as the main axis.

## 4. Feature Extractions

In this work, three different feature extractions method were used to obtain relevant identifiers from the PD data; they are statistical features, fractal features and principal component analysis (PCA) features. These features are chosen because they are the most commonly used features in PD classification. They are then combined together to enhance the performance of classifiers using multiple features instead of individual features. These input features were used to train the ANN, ANFIS, and SVM to classify defect types.

### 4.1 Statistical Features

PD data can be characterized by two main distributions; pulse count distribution, which is the number of PD vs. phase angle and pulse height distribution, which is the PD charge magnitude vs. phase angle. These distributions can be further split into two separate distributions, which are the negative and positive half cycles. Statistical features were extracted from these PD distributions, which include skewness, kurtosis, mean, variance and the Weibull parameter.

Skewness is the degree of asymmetry of the distribution with regard to the normal distribution. Positive skewness shows that the distribution is asymmetric with a bigger left side, zero skewness shows that the distribution is symmetric and negative skewness shows that the distribution is asymmetric with a larger right side [44].

Kurtosis is the degree of the sharpness of the distribution with regard to a normal distribution. Zero kurtosis shows that the distribution is a normal shape, positive kurtosis shows that the distribution is a sharp shape and negative kurtosis shows that the distribution is flat shape [45].

Variance is a measurement of how much a cluster of numbers is spread out. Zero variance shows that all values in the distribution are identical. The standard deviation is acquired by calculating the square root of the variance. A very detailed mathematical description of skewness and kurtosis can be found in [46]. The mean, variance, skewness and kurtosis are calculated using

$$\text{Mean}:\mu =\frac{\sum_{i=1}^{N}x_{i}f\text{(}x_{i}\text{)}}{\sum_{i=1}^{N}f\text{(}x_{i}\text{)}}$$ (1)

$$\text{Variance}:\sigma^{\text{2}}=\frac{\sum_{i=1}^{N}{\text{(}x_{i}-\mu \text{)}}^{2}f\text{(}x_{i}\text{)}}{\sum_{i=1}^{N}f\text{(}x_{i}\text{)}}$$ (2)

$$\text{Skewness}:S_{k}=\frac{\sum_{i=1}^{N}{\text{(}x_{i}-\mu \text{)}}^{3}f\text{(}x_{i}\text{)}}{\sigma^{3}\sum_{i=1}^{N}f\text{(}x_{i}\text{)}}$$ (3)

$$\text{Kurtosis}:K_{u}=\frac{\sum_{i=1}^{N}{\text{(}x_{i}-\mu \text{)}}^{4}f\text{(}x_{i}\text{)}}{\sigma^{4}\sum_{i=1}^{N}f\text{(}x_{i}\text{)}}-3$$ (4)

where *f*(*x*_*i*_) is the function of interest, *N* is the size of the data and *x*_*i*_ is discrete values of the distribution.

Weibull analysis is a mathematical approach to characterize the pulse height analysis pattern. The probability distribution of PD pulse rate, *F* can be expressed by the Weibull function [20, 47] as

$$F(q;\alpha ;\beta )=1-\text{exp}\left[-{\left(\frac{q}{\alpha }\right)}^{\beta }\right]$$ (5)

where *α* and *β* represents each pulse height analysis curve and the PD pulse amplitude is represented by *q*. The features *α*+, *β*+, *α*- and *β*- are obtained from the negative and positive pulse height analysis curves [20]. The pulse height analysis pattern is then compacted using the Weibull method for statistical analysis while keeping its relevant information. The values of *α*+, *β*+, *α*- and *β*- are then used as the input to the intelligent classifiers along with variance, skewness, kurtosis and mean.

### 4.2 Fractal Features

Fractal features are suitable for modeling complex shapes and natural phenomena where current mathematical methods are found to be inadequate. Since PD can be treated as a natural phenomenon that has complicated shapes and surfaces, fractal features can be used to model it. The implementation of fractal features in PD recognition is interesting because it characterizes the PRPD pattern directly [48].

Fractal features can also be used for pattern recognition [49]. PRPD pattern can be characterized using two fractal features, fractal dimension and lacunarity, which are computed by using box counting technique. Fractal dimension is one of the main fractal features that could be computed from an image surface. In theory, fractal dimension is invariant to changes in scale and has the potential to be used for measuring the coarseness of the surface. However, fractal dimension alone is not enough to be a discriminatory feature because different surface may have the exact same value of fractal dimension. In order to solve this problem, Mandelbrot has introduced a new variable called lacunarity, which represents the compactness of the fractal surface. Both fractal dimension (*D*) and lacunarity (*Λ*) are functions of the box size *L*. The number of boxes *N*, of side *L* needed to cover a fractal set is governed by

$$N(L)=KL^{-D}$$ (6)

where *D* is the fractal dimension set and *K* is a constant [50]. The lacunarity *Λ*(*L*) relies on the second order statistics of *p*(*m*,*L*). It can be defined after calculating *M*(*L*) and *M*_*2*_(*L*). *Λ*(*L*), *M*(*L*) and *M*_*2*_(*L*) are calculated using [51]

$$\wedge (L)=\frac{M^{2}(L)-{\left[M(L)\right]}^{2}}{{\left[M(L)\right]}^{2}}$$ (7)

$$M(L)=\sum_{m=1}^{N}mp(m,L)$$ (8)

$$M^{2}(L)=\sum_{m=1}^{N}m^{2}p(m,L)$$ (9)

where *m* is the box number. In this work, PRPD patterns were converted into a binary image and the software ImageJ was used to calculate the fractal dimension and lacunarity using the box counting method [52].

### 4.3 Principal Component Analysis

PCA, also known as the Karhunen-Loève (K-L) method is a data reduction method that can filter out the important factors from a big group of data [53]. It is able to transform the data from a very high dimension to a lower dimension. This is done without compromising data information in the reduced space, with only minimal information loss. This is achieved by projecting data at a direction with the biggest variance at a lower dimension that will maximize the scatter of the projected samples [54]. This linear subspace is found by solving an Eigen problem,

cov(X)M=λM (10)

where *cov*(*X*) is the covariance matrix of the dataset *X*, *M* is a linear mapping created by the *d* principle eigenvectors of the covariance matrix and *λ* are the *d* principal eigenvalues. The low-dimensional data *y*_*i*_ of the data points *x*_*i*_ are calculated using linear mapping *Y = XM*. The elements of *Y* will produce the feature sets [17]. This covariance matrix is able to determine which direction contains the most significant variance in the dataset, making PCA an effective tool for feature subset selection.

The most important concern in PCA is the amount of principal components required to obtain an accurate representation of the original data. The best number of principal components to represent the data can be found by using a scree plot. Scree plot is a graph of the eigenvalue magnitude vs. its number. The best number is chosen at a point where the graph has a sudden change in a slope, where the slope on its left side is much higher than the right side. [53].

The PD data were arranged into 3 column matrix of phase, magnitude and pulse count which is similar to the PRPD format. Two situations were considered for PCA feature extraction. In the first situation, the PD matrix was split into four distributions, negative and positive section of the charge magnitude while the phase was divided into two 180 degrees’ quadrants. In the second situation, the PD matrix was arranged into six distributions, negative and positive section of the charge magnitude while the phase was divided into four 90 degrees’ quadrants. PCA was performed on these distributions to obtain the first and second principal components.

## 5. Classifiers

Three intelligent classifiers were used; they are Adaptive Neuro-Fuzzy Inference System (ANFIS), Artificial Neural Network (ANN) and Support Vector Machine (SVM). These classifiers were trained and then used to classify defect types of the cable joint samples in this work.

### 5.1 Artificial Neural Network (ANN)

ANN is suitable for PD classification because it is insensitive to small input changes. ANN has the ability to continue making correct decisions even when the input presented is slightly different from the input used during training process. This is very important for PD classification where the discharge patterns are usually not the same [55].

ANN consists of one layer of input, a minimum of one hidden layer and one output layer [1, 56]. The feed forward back propagation neural network is the most commonly used learning mode in ANN [57]. It is a supervised learning network that is trained in a forward backward process. In the forward process, the biases and weights are initialized into random small values. The feature vector that belongs to its correlating sample is then used to compute the neurons output in each layer using an activation function that can be threshold using different functions [12].

Every layer in the ANN is completely connected to the following layer. The main purpose of the hidden layer is to obtain PD features from different sources and send the information to the output layer. The amount of processing elements in the input layer relies on the amount of PD fingerprint data. The amount of processing element within the output layer is dependent on the number of defect types to be classified [45]. For PD classification purpose, a minimum of two input features are required to avoid divergence during training [58]. In this work, a multilayer feed forward ANN with 15 neurons at the hidden layer and the scaled conjugate gradient back propagation training function were used. Sigmoid function was used as the activation function at the hidden layer and output layer.

### 5.2 Adaptive Neuro-Fuzzy Inference System (ANFIS)

ANFIS uses neural networks and fuzzy systems to find the best fuzzy parameters [59]. The usage of neural network omits the requirement to select the fuzzy parameters manually because it will be done by the neural network. The fuzzy system must be built using fuzzy logic prior to the fuzzy scheme training in ANFIS. ANFIS is based on a fuzzy Sugeno model introduced by Takagi, Sugeno and Kang. ANFIS is a great tool to map PD patterns to the defect type using If-Then rules formed by the decision tree and the stipulated input output data [60].

$$\text{Rule }1:\text{ If }x\text{ is }C_{1}\text{ and }y\text{ is }D_{1},\text{ then }f_{1}=p_{1}x+q_{1}y+r_{1},$$

$$\text{Rule }2:\text{ If }x\text{ is }C_{2}\text{ and }y\text{ is }D_{2},\text{ then }f_{2}=p_{2}x+q_{2}y+r_{2}.$$

The ANFIS architecture has five important layers [61]. The first layer is filled with nodes called adaptive nodes. The outputs of this layer are known as the fuzzy membership grade of the inputs. In the second layer, it contains constant nodes that function as a multiplier for incoming signal. The output of this layer is called the firing strength of the rules. The third layer contains fixed nodes, which concentrates on normalizing the second layer’s triggering strength. In the fourth layer, the nodes are adaptive nodes. It will produce output which is the product of the first order polynomial and the firing strength that had been normalized. The last layer contains only one fixed node, which does a summation of all output signals from the previous layer.

Rules fuzzification is done by allocating fuzzy membership function (MF) to each condition in the premise part of the rules. Each input variable is normalized between zero and one in order to increase the training efficiency [62]. Utilizing these fuzzy rules, ANFIS is used to train, test and analyze the Sugeno-type fuzzy inference system [63]. Every rule output works as a linear combination of input variables and a fixed value. The final output is the output weighted mean of each rule. These weights are automatically altered using the information acquired during the training process. For ANFIS, Matlab command “genfis2” was used to generate a Sugeno type fuzzy inference system using subtractive clustering. “Genfis2” was used instead of “genfis1” since it is more suitable for large amount of data used in this work. The ANFIS used has 20 “epoc” and 1 “radii,” where “epoc” is the maximum number of times before the training process is stopped. “Radii” is a vector that specifies a cluster center range of influence in each data dimensions, assuming the data falls within a unit hyperbox.

### 5.3 Support Vector Machine

SVM is a machine learning algorithm that stem from statistical learning theory. This learning machine uses a main concept of SVM, which is kernel for a variety of learning tasks. Using kernel methods, SVMs can be adjusted to multiple types of tasks by using different base algorithm and kernel functions. SVM excels in pattern recognition problems involving nonlinear, small sample size and high dimensionality [64].

SVM is a method for searching functions from a group of known as training data. Individual group of PD pattern data can be characterized by specific input features. Therefore, each group of data can be designated by a vector whose size and dimension relies on the number of input features selected to characterize it. The function can be either a regression function or a classification function. It is commonly utilized to process classification and regression problems.

SVM is initially intended to handle linearly separable cases. Unfortunately, not all practical problems are linearly separated. When dealing with non-linear problems, conventional SVM as a linear classifier will not function effectively. To overcome this problem, a technique known as kernel was presented to deal with non-linear problems using multiple linear classifier. According to the pattern recognition theory, a lower dimensional space and non-linear inseparable model are transformed into linear separable by mapping it nonlinearly into a higher dimensional feature space. Therefore, the usage of kernel method will avoid the curse of dimensionality [15].

SVM algorithm was initially intended for binary classification, which means they can only classify inputs into two classes [65]. This is because SVM uses a hyper plane to split data into two categories. If more than two groups of classification are required, multi-level SVM is needed [66, 67]. Multi-level SVM is a one against all classifiers, where multiple binary SVM is performed. During multi-level SVM training, a category sample will be classified as one class while the other residual samples as other classes. In this work, a multilevel SVM with the radial basis function kernel was used as the classifier.

## 6. Results

The measurement results from this study are shown in this section. The results include the PD patterns measured and classification accuracy results of ANN, ANFIS, and SVM using statistical, fractal and PCA features. Next, the noise tolerance of each classifier was examined.

### 6.1 Measured PD in PRPD format

In order to determine the classification accuracy of each feature extraction and intelligence classifier method under noisy environment, the classifiers are trained using uncontaminated input but tested with inputs that are contaminated with noise. Feature extractions are performed on the contaminated PD data and used as the test input to each classifier method. The noise contamination is recorded from interference from the ground during lightning events. The recorded noisy signals are added to the noise-free PD data for duration of between 5 and 60 seconds.

**Fig 2:**
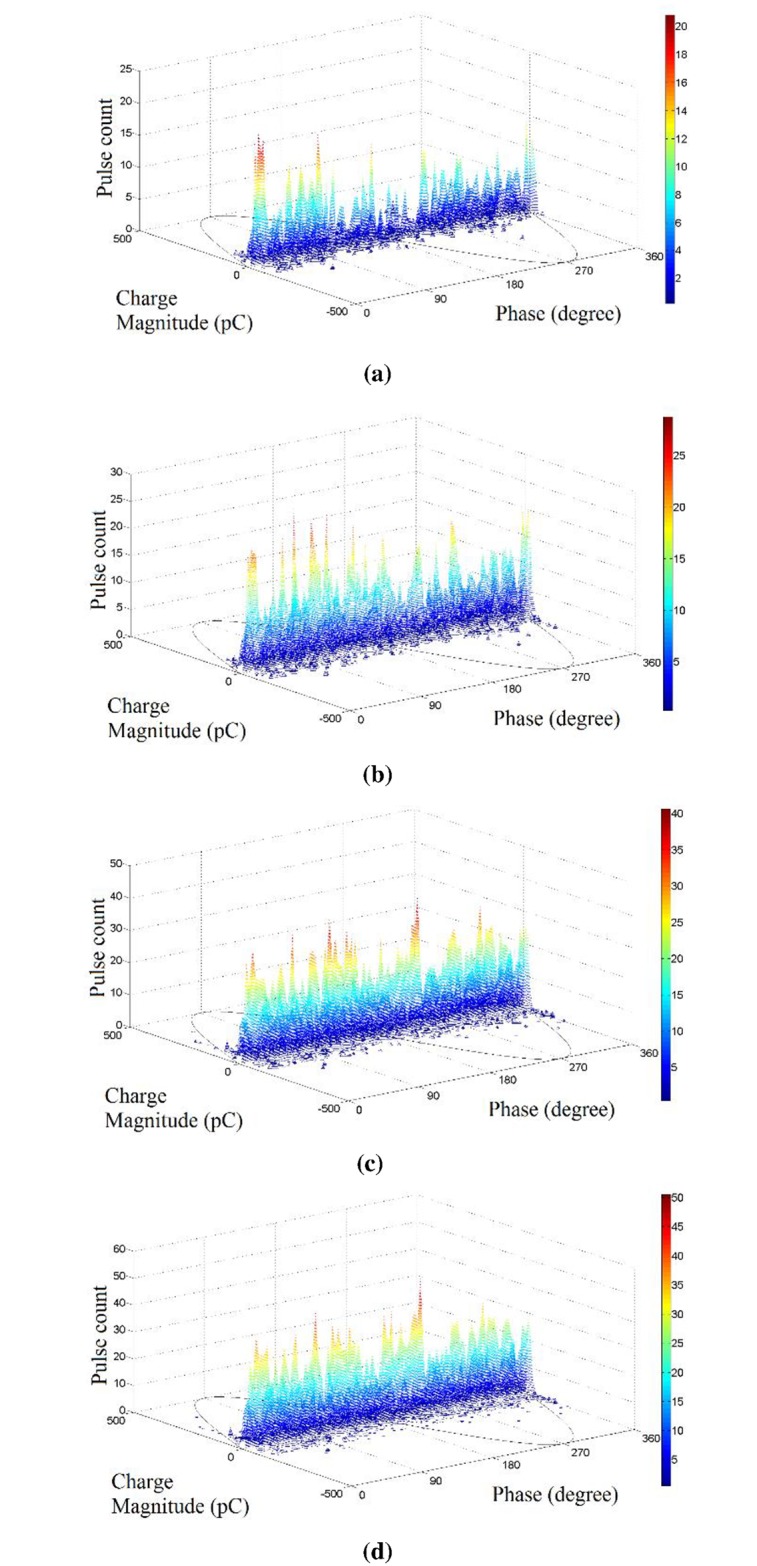
PRPD patterns of different noise duration; (a) 15 seconds, (b) 30 seconds, (c), 45 seconds, and (d) 60 seconds.

Four of the PRPD patterns of the recorded noisy signal are shown in Fig 2. It can be seen that the noise pattern occurs randomly at every phase with a maximum amplitude of 250 pC and the number of PD activity increases as the duration of noise increases. Since it is impossible to control the amplitude of the noise contamination, different duration of the noise contamination was used to examine the classification accuracy of each classifier methods under noisy environment.

**Fig 3:**
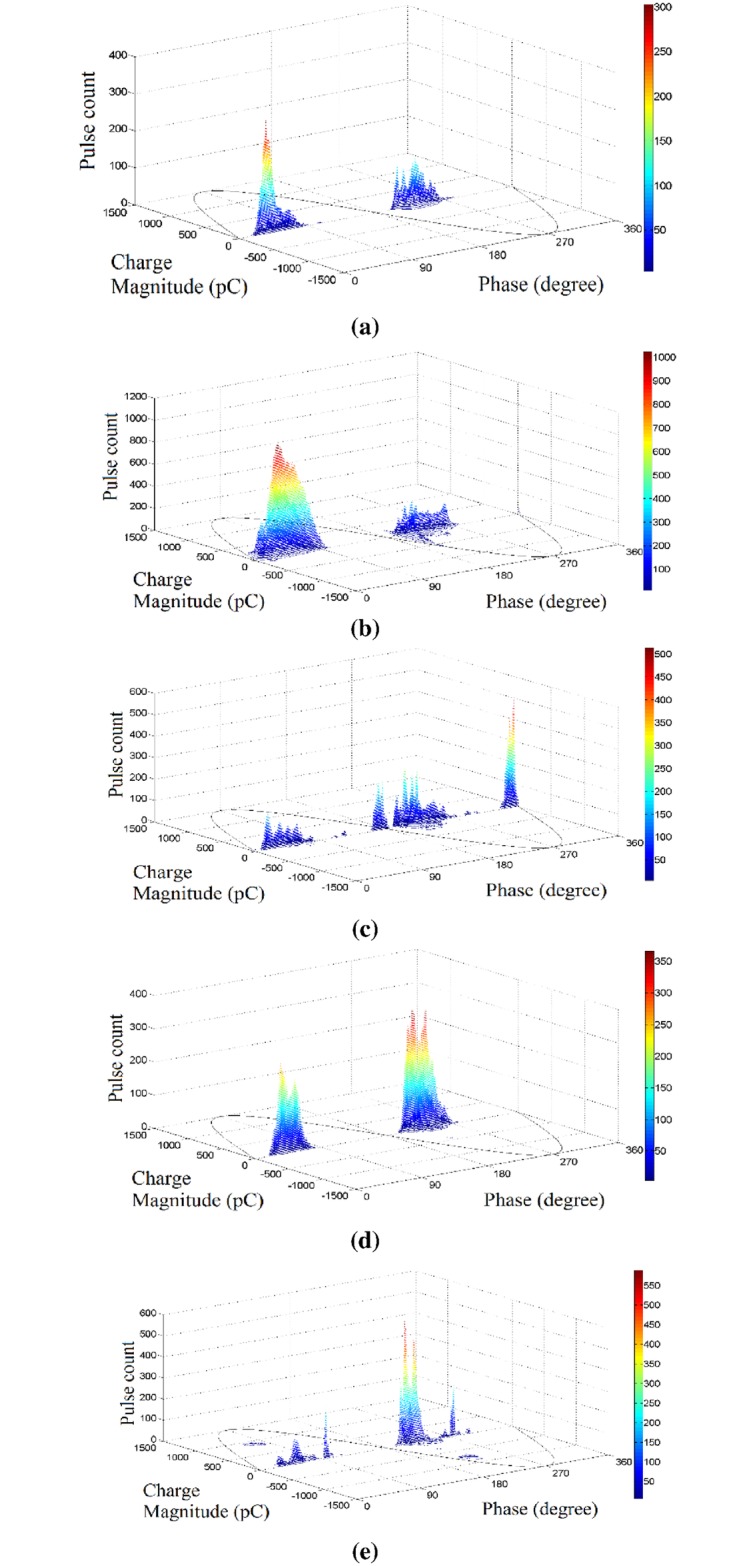
PRPD patterns from samples of different defects; (a) Insulation incision defect (b) axial direction shift defect, (c), semiconductor layer tip defect (d) metal particle on XLPE defect and (e) semiconductor layer air gap defect.

The phase resolved partial discharge (PRPD) pattern of all cable joint samples that have been measured is shown in a 3D plot in Fig 3. Based on visual inspection on the PRPD patterns, the insulation incision defect has two tall peaks at the end of both positive and negative cycles. The axial directional shift defect has more PD activities in the positive cycle, which accumulate at the first quadrant. It has a very sharp peak at around 80 degrees. The semiconductor layer tip defect has PD activities, which extend evenly between the positive and negative cycles. It has 5 noticeable peaks, 3 at the negative cycle and 2 at the positive cycle. The metal particle on XLPE defect has one main PD group at each positive and negative cycles and it has a prominent peak at 260 degrees. The semiconductor layer air gap defect has two main PD groups; one at the positive cycle and another at the negative cycle with a peak at 230 degrees. Two small clusters of PD with high charge magnitude but low pulse count can also be seen, where the negatively charged PD spread out between 180 to 360 degrees while the positively charged PDs are distributed between 0 and 180 degrees.

Although different defect types of cable joint have different PRPD patterns, classification of different defect types in the cable joint samples can be hardly done based on visual inspection on these PRPD patterns alone. Therefore, feature extractions from PD data and intelligent classifiers were used in this work to classify different defect types in the cable joint.

### 6.2 Feature extraction results

Using the feature extraction method of statistical features, fractal features and PCA features, eight groups of input feature data were obtained. Statistical features are split into three groups, the first group consist of variance, skewness, kurtosis and mean (var, skew, kur, mean), the second group consists of Weibull parameters while the third group is the combination of the first two groups. Fractal features were also split into three groups, which are fractal dimensions, lacunarity and a combination of fractal dimensions and lacunarity. PCA features were split into two groups and PCA features from 4 and 6 distributions are extracted. These input features were used as the input for the classifiers to determine the classification accuracy of each method. Sample input features extracted using each method are shown in Tables 2, 3 and 4.

**Table 2:** Extracted statistical features.

Sample	C1	C2	C3	C4	C5
Mean *H*_*n*_^+^*(φ)*	41.2944	548.2333	73.8111	45.3944	35.6944
Mean *H*_*n*_^-^*(φ)*	43.3833	293.5556	107.9278	108.9667	52.8833
Stdev *H*_*n*_^+^*(φ)*	78.4387	690.5267	105.5642	83.9630	46.7474
Stdev *H*_*n*_^-^*(φ)*	69.3773	384.3038	147.7483	184.3328	81.0928
Skewness *H*_*n*_^+^*(φ)*	2.2340	0.7831	1.5270	1.7639	1.1117
Skewness *H*_*n*_^-^*(φ)*	1.4984	0.9107	1.6990	1.5827	2.0038
Kurtosis *H*_*n*_^+^*(φ)*	7.1230	1.9383	4.1013	4.7041	2.8598
Kurtosis *H*_*n*_^-^*(φ)*	3.9166	2.3737	5.2176	4.1422	6.5342
Mean *H*_*qn*_^+^*(φ)*	29.8984	60.3932	36.7744	24.8165	239.9170
Mean *H*_*qn*_^-^*(φ)*	-46.9201	-70.9458	-50.7191	-33.0217	-168.3107
Stdev *H*_*qn*_^+^*(φ)*	14.8031	355.9565	60.3246	44.6759	390.1542
Stdev *H*_*qn*_^-^*(φ)*	32.0612	231.3776	72.4628	47.5718	337.4364
Skewness *H*_*qn*_^+^*(φ)*	1.3793	16.4857	5.3307	8.0583	1.5959
Skewness *H*_*qn*_^-^*(φ)*	-0.7037	-25.4588	-5.8096	-6.9205	-2.4466
Kurtosis *H*_*qn*_^+^*(φ)*	8.2998	278.1250	42.6534	78.3225	3.9256
Kurtosis *H*_*qn*_^-^*(φ)*	2.5962	722.1208	76.0322	68.9398	7.5861
Weibull *α+ H*_*qn*_^+^*(φ)*	33.8424	47.5005	49.1166	45.9543	54.6889
Weibull *β+ H*_*qn*_^+^*(φ)*	2.1356	0.8096	1.0378	1.0411	0.8202
Weibull *α- H*_*qn*_^-^*(φ)*	52.2874	67.0304	58.0309	55.3513	60.2303
Weibull *β*- *H*_*qn*_^-^*(φ)*	1.5167	0.9212	0.9392	0.9421	0.8430

**Table 3:** Extracted PCA features.

Sample	C1	C2	C3	C4	C5
1st Principal component	15466.92	53812.19	28191.27	22645.52	24781.90
	-10023.63	-86101.03	-18434.80	-13376.07	-36949.43
	-5443.29	32288.85	-9756.46	-9269.45	12167.53
2nd Principal component	396.12	23468.59	-1368.24	-513.11	9458.52
	1808.37	4266.60	-5982.90	-3987.69	2429.16
	-2204.49	-27735.19	7351.14	4500.80	-11887.68
Latent	184664096	5675854466	614888988	388830821	1063725994
	4143434	669109687	45853246	18211088	118340647

**Table 4:** Extracted fractal features.

Sample	C1	C2	C3	C4	C5
Fractal dimensions	1.0194	1.1832	1.2625	1.1153	1.4403
	1.0216	1.2099	1.2707	1.1323	1.4284
	1.0165	1.2239	1.2939	1.1385	1.4472
	1.0312	1.1872	1.2455	1.0955	1.4237
	0.9945	1.1593	1.2234	1.0693	1.3844
	0.9807	1.1614	1.2184	1.0679	1.3809
	0.9745	1.1518	1.2207	1.0687	1.3734
	0.9800	1.1530	1.2161	1.0611	1.3819
	0.9801	1.1496	1.2143	1.0539	1.3809
	0.9793	1.1584	1.2269	1.0711	1.3822
	0.9774	1.1593	1.2245	1.0652	1.3759
	0.9754	1.1524	1.2124	1.0641	1.3757
Lacunarity	3.2030	2.8662	3.6734	4.3090	2.1689
	3.1826	2.7053	3.6556	4.2790	2.1232
	3.1259	2.6616	3.4191	4.1019	2.1006
	3.1382	2.7285	3.7433	4.4175	2.1653
	3.3055	2.9040	3.9493	4.7882	2.3764
	3.5426	2.8755	3.9130	4.7669	2.3790
	3.4176	2.8940	3.8376	4.6951	2.4244
	3.5118	2.9119	3.8289	4.7892	2.4159
	3.3419	2.8563	3.7805	4.5176	2.3105
	3.5261	2.9234	3.9009	4.7871	2.3782
	3.4471	2.8645	3.8581	4.7480	2.4381
	3.4664	2.7786	3.8659	4.7270	2.4359

### 6.3 Classification results

In this work, 10-fold cross validation method was used. The data was randomly partitioned into 10 equal sized subsamples. One subsample was used for testing and the remaining nine subsamples were used for training. The process was repeated for a total of 10 times with each subsample taking turns to be the test sample and the mean accuracy was calculated. The classification results of ANN, ANFIS and SVM using different feature extraction methods are shown in Table 5.

**Table 5:** Classification accuracy results using noise-free PD data.

Classifier	Input type	Cable joint default type					Total
		C1	C2	C3	C4	C5	
ANN	Var, skew, kur, mean	90	98	86	85	93	90.4
	Weibull Parameters	96	80	73	82	91	84.4
	Statistical features	95	88	90	93	100	**93.2**
	Fractal dimensions	78	77	54	59	89	71.4
	Lacunarity	80	96	90	78	91	87.0
	Fractal features	88	87	88	78	100	**88.2**
	PCA (4 distributions)	89	95	72	84	93	86.6
	PCA (6 distributions)	90	91	82	88	95	**89.2**
ANFIS	Var, skew, kur, mean	96	96	99	92	100	96.6
	Weibull Parameters	92	24	90	89	92	77.4
	Statistical features	96	95	99	95	100	**97.0**
	Fractal dimensions	42	89	76	58	82	69.4
	Lacunarity	47	95	90	69	96	79.4
	Fractal features	73	99	86	67	100	**85.0**
	PCA (4 distributions)	43	86	71	55	88	68.6
	PCA (6 distributions)	61	86	74	66	90	**75.4**
SVM	Var, skew, kur, mean	99	99	98	87	100	96.6
	Weibull Parameters	100	57	95	0	89	68.2
	Statistical features	100	99	98	97	100	**98.8**
	Fractal dimensions	95	94	69	19	97	74.8
	Lacunarity	93	100	86	78	98	91.0
	Fractal features	95	99	94	75	100	**92.6**
	PCA (4 distributions)	96	97	89	48	98	85.6
	PCA (6 distributions)	98	96	93	85	100	**94.4**

From Table 5, SVM has the highest overall classification accuracy. ANFIS performed better than ANN when using statistical features but is the worst when using fractal features and PCA features. ANFIS is weak when using PCA features because ANFIS requires normalizing the input data during the training process to improve its efficiency [63]. PCA component contains of a different weighting; hence normalization will change the relative significance between each components, causing higher error rate in ANFIS [68].

It can be seen that for all three classifiers, using the combination of all statistical features and fractal features rather than splitting them results in higher classification accuracy. For PCA features, all classifiers are able to achieve higher classification accuracy when PCA is performed on 6 distributions instead of 4 distributions. Therefore, for classification accuracy test using noisy signals, only the full set of statistical parameters (variance, skewness, kurtosis, mean, Weibull parameters), fractal features (fractal dimension and lacunarity) and PCA features for 6 distributions were considered.

**Fig 4:**
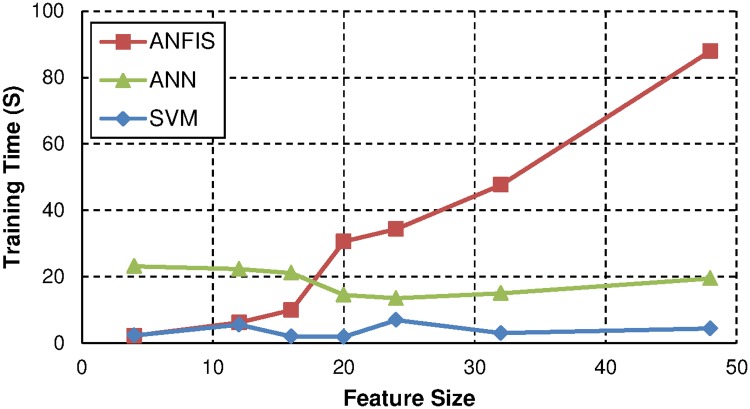
Training time vs feature size for PD classifiers.

The effect of increasing feature size on the training duration for all classifiers is shown in Fig 4. From this figure, SVM has the fastest training speed, followed by ANN and ANFIS. SVM and ANN training speed is not directly affected by the size of the input feature and remains relatively consistent when the feature size is increased. ANFIS, on the other hand, experiences increased training duration when the input feature size is increased.

After the performance of ANN, ANFIS and SVM with noise-free PD data has been evaluated, the classifiers were tested using features extracted from PD data that have been overlapped with noise contamination of different durations. The classification accuracy results of these classifiers are shown in Table 6. From Table 6, it can be seen that the classification accuracy generally decreases for all classifiers and input feature combinations when more noise is added.

**Table 6:** Classification accuracy results using PD data added with noise.

Classifier	Input type	Duration of noise contamination (s)											
		5	10	15	20	25	30	35	40	45	50	55	60
ANN	Statistical	91.8	92.6	85.2	75.6	67.6	62.6	49.8	50.6	44.4	45.4	42.4	42.0
	Fractal	62.6	60.0	44.8	40.8	36.2	34.8	30.2	28.4	30	27.4	30.8	28.0
	PCA	89.0	89.0	88.0	89.2	84.2	81.4	78.2	73.2	71.4	70.2	70.4	70.4
SVM	Statistical	91.6	95.2	80.4	55.8	58.2	45.8	49.4	42.8	51	43	52.2	48.2
	Fractal	69.8	69.4	65.4	65.0	64.4	62.8	60.8	59.8	59.0	58.2	58.0	58.4
	PCA	93.6	93.0	89.6	89.2	82.8	81.6	77.8	77.0	74.8	74.6	76.2	75.6
ANFIS	Statistical	91.4	92.8	61.4	54.8	56.2	46.8	26.2	23.6	22.4	20.6	16.8	21.4
	Fractal	20.6	21.6	19.0	23.2	22.4	20.4	19.6	20.8	20.0	19.8	20.4	19.8
	PCA	73.4	73.8	68.0	62.0	60.0	64.4	67.4	64.4	66.4	57.6	58.2	53.0

**Fig 5:**
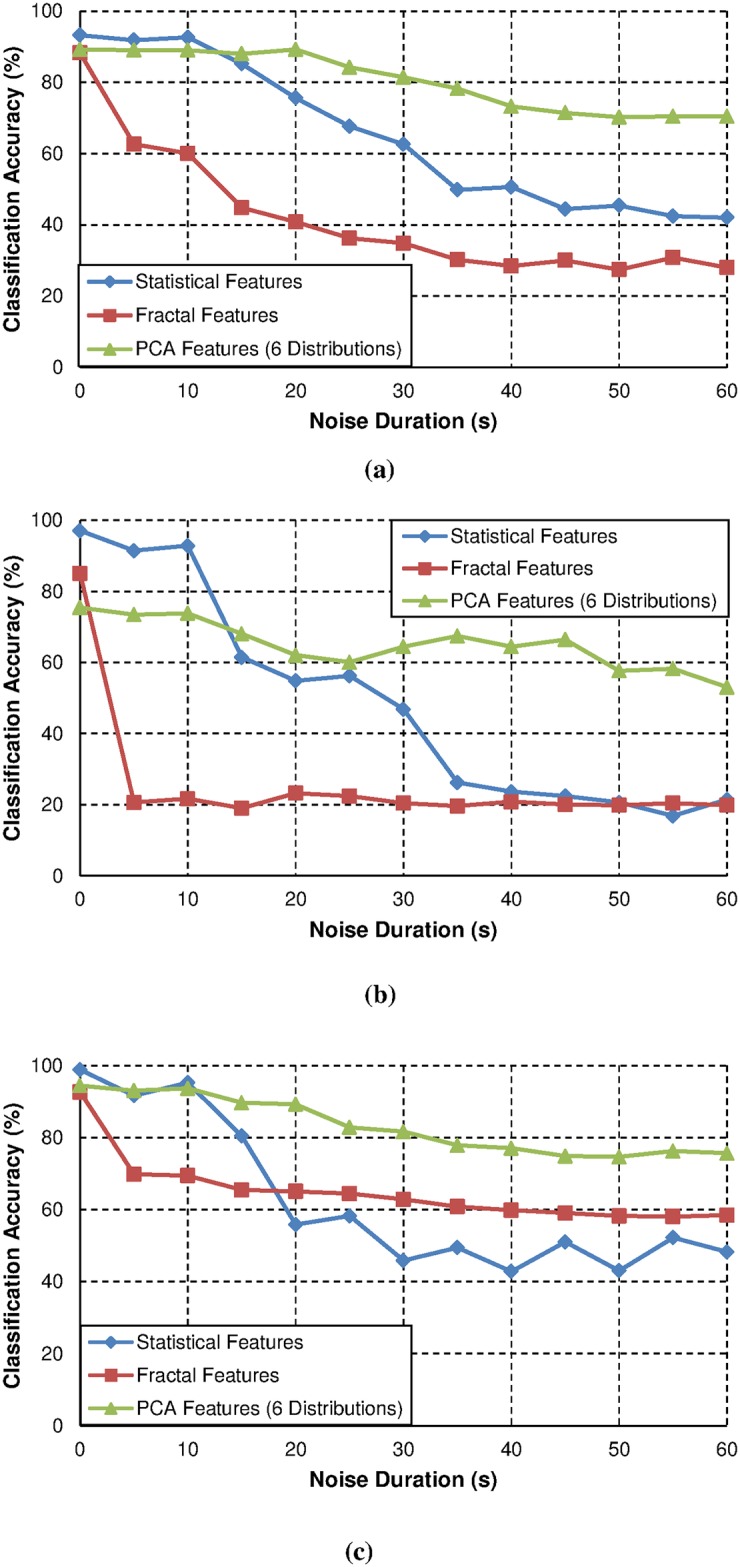
Noise tolerance of; (a) ANN, (b) ANFIS, and (c) SVM.

The plot of classification accuracy against the duration of noise contamination added to PD data for all classifiers is shown in Fig 5. From this figure, it can be seen that although statistical features and fractal features suffer from significant reduction in classification accuracy, statistical features still achieve higher classification rate than fractal features for ANN and ANFIS when the noise duration is increased. ANFIS performs slightly better with fractal features as the input was used in noise-free PD data but its performance with the statistical features is better than fractal features.

When each classifier was tested with noisy PD data, classifier with PCA features as the input data performs better than with fractal features and statistical features. Although the classification accuracy by using PCA features is not the best for noise-free PD data for all classifiers, the classification accuracy is better than other feature extractions when being tested with noisy data. This is due to the changes to the original PD data due to noise are minimized during the process of transforming the PD data from a higher dimension to a lower dimension. Thus, this causes classification accuracy using PCA features and intelligent classifiers to be less affected by different durations of noise contamination compared to statistical and fractal features.

Referring to Table 6, the best classification method is PCA combined with SVM, where the highest classification accuracy is 93.6% under 5-second noise duration and 75.6% under 60-second noise duration. Comparing with previous works in [1], the accuracy of ANN reduces from 79% under noise free condition to 42.2% with 10% added noise while in [34, 38], when 30% noise was introduced, the accuracy of ANN reduces from 100% to between 70 and 80%. Hence, this shows that the proposed method in this work is reasonable and has an improvement over the previous methods used for PD classification under noisy condition. This is due the classification accuracy reduction is smaller than the previous works when noise contamination was added to PD signals.

## 7. Conclusions

Classifications of real cable joint defect types from partial discharge measurement under noisy environment have been successfully performed. Feature extractions were performed on the PD data and used as the input data for artificial intelligence classifiers to classify cable joint defect types. From the classification accuracy results, feature extraction using principal component analysis (PCA) features and Artificial Neural Networks (ANN) and Support Vector Machine (SVM) classifiers show the highest classification accuracy when being tested with noisy PD data. Adaptive Neuro-Fuzzy Inference System (ANFIS) classifier is not suitable to be used with PCA features due to the design of the classifier which requires normalization during training. Classification accuracy by using feature extractions of fractal features and statistical features with the classifiers is better than using PCA features for noise-free PD data but is worse for noisy PD data. If computational time is not an important factor, it is recommended that the three input features (include statistical features, fractal features and principal component analysis) are used together to complement each other. However, if only one type of classifier and input feature is to be used in a highly noisy environment, PCA features and SVM or ANN is recommended for PD classification.
